# Evidence for PDGFRA, PDGFRB and KIT deregulation in an NSCLC patient

**DOI:** 10.1038/sj.bjc.6603542

**Published:** 2007-01-09

**Authors:** T Negri, P Casieri, F Miselli, M Orsenigo, C Piacenza, S Stacchiotti, P Bidoli, P G Casali, M A Pierotti, E Tamborini, S Pilotti

**Affiliations:** 1 Laboratory of Experimental Molecular Pathology, IRCCS Istituto Nazionale dei Tumori, Via Venezian, 1 20133 Milan, Italy; 2 Department of Medical Oncology, IRCCS Istituto Nazionale dei Tumori, Via Venezian, 1 20133 Milan, Italy; 3 IRCCS Istituto Nazionale dei Tumori, Via Venezian, 1 20133 Milan, Italy

**Sir**,

Vlahovic *et al*, provided evidence of effectiveness of Imatinib treatment in PDGFRB-overexpressing human lung adenocarcinoma xenografts grown in nude mice. In this preclinical model, the effectiveness of the drug is related to decrease in interstitial fluid pressure (IFP), decrease of phosphorylated PDGFRB and VEGF expression and improvement of oxygenation (Vlahovic *et al*, 2006). Furthermore, according to the working hypothesis that inhibition of PDGFRB decreases IFP and improves first-line therapy through drug uptake increase (Pietras, 2004), a clinical trial investigating the role of Imatinib as an adjuvant to chemotherapy is ongoing in stromal PDGFRB expressing non-small-cell lung cancer (NSCLC) (Vlahovic *et al*, 2006). Here we demonstrate that Imatinib in adjunction to chemotherapy may also be effective in a lung adenocarcinoma made up of tumour cells expressing Imatinib-sensitive deregulated genes such as *PDGFRA*, *PDGFRB* and *c-Kit* in presence of *EGFR* and *HER-2/neu* amplification lacking any evidence of protein expression.

An NSCLC, corresponding to a poorly differentiated adenocarcinoma, was diagnosed 1 month after starting treatment with Imatinib for a locally advanced sacral chordoma in a 64-year-old male patient. The chordoma expressed an activated PDGFRB (detected by immunoprecipitation/Western blotting (IP/WB) experiments) and the cognate ligand (by RT–PCR), findings consistent with the presence of an autocrine/paracrine loop (Casali *et al*, 2004). At the time the second primary was detected, the chordoma was showing a weak dimensional response to Imatinib.

The lung carcinoma was initially surgically treated, but relapsed to the mediastinum 17 months later, when the chordoma seemed to undergo slight secondary progression on Imatinib. Soon after stopping Imatinib, the patient received cisplatin plus vinorelbine with concomitant radiotherapy and, four months later, at the end of chemo-radiotherapy, CT and PET revealed an optimal complete radiological and functional response of lung tumour. Surprisingly, given the poor chemosensitivity of chordomas, there was also an initial minor response of the sacral tumour, which was lost soon after, but then was restored with the resumption of Imatinib (the plan was to reintroduce the latter in combination with cisplatin, but renal function did not allow it).

The primary lung tumour was investigated in addition to FISH analysis, by IHC, IP/WB and DNA sequencing (after the informed consent of the patient) (Figure 1). Cumulatively, the FISH findings were consistent with a high degree of genetic instability showing high polisomy of *PDGFRA*, *PDGFRB* and *c-Kit* genes, coupled with activation (phosphorylation) of all the three receptors at IP/WB analysis and positivity at IHC (anti-PDGFRA sc-338 and anti-PDGFRB sc-339, Santa Cruz Biotechnology, CA, USA; anti-CD117 A4502, Dako, Carpinteria, CA, USA), as well as, as already anticipated, *EGFR* and *HER-2/neu* amplification coupled with null IHC (Dako) and biochemical analysis. Molecular analysis performed for investigating the mutation hot-spots of RTK genes excluded the presence of any activating mutation.

Finally, FISH analysis performed on the chordoma biopsy obtained during surgery for adenocarcinoma showed a dysomic pattern of all the investigated RTK genes.

The present findings imply firstly that the lung adenocarcinoma tumoral cells, in addition to *EGFR* and *HER-2/neu* gene deregulation, successfully treated with EGFR family inhibitors (Bunn *et al*, 2006), may harbour gene amplification and phosphorylation of Imatinib-sensitive RTKs, and so the spectrum of deregulated RTK genes in NSCLC is wider than believed. Secondly that, although supported by different mechanisms (an autocrine/paracrine activation loop of PDGFRA, PDGFRB and KIT in chordoma (Tamborini *et al*, 2006) and a gene gain in lung cancer), the two tumours carried the same Imatinib-sensitive deregulated genes and are thus expected to be sensitive to the same drug. The sequence of clinical events is hard to interpret but, together with the biomolecular findings, what was observed strongly supports the possibility that the efficacy of the chemotherapy against the NSCLC may have benefited from Imatinib, and the efficacy of Imatinib against chordoma may have benefited from the chemotherapy. Cumulatively, all these findings give further support to the ongoing clinical trial in selected patients with NSCLC (Vlahovic *et al*, 2006) and, in particular, for PDGFRB, they point out that this receptor is a multifaced player that through different mechanisms may affect the treatment response of both standard treatments and target therapies.

## Figures and Tables

**Figure 1:**
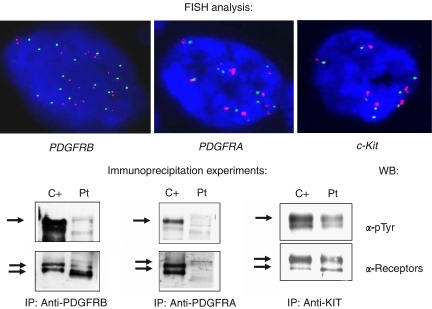
Upper panel. FISH analysis. A probe mix for *PDGFRB* (BAC clone RP11-368O19) (in green)/a locus specific in 5p (in red), *PDGFRA* (BAC clone RP11-231C18) (in green)/Cep4 (in red) (Vysis) and *c-Kit* (BAC clone RP11-586A2 (in green)/Cep4 (in red) (Vysis), respectively, was employed to detect gene status for the receptors. A condition of high polisomy was detected for *PDGFRB*, *PDGFRA* and *c-Kit*. Lower panel. Immunoprecipitations/Western blot analysis. Equal amounts of total protein extracts (1 mg) were immunoprecipitated using the specified antibodies (PDGFRB sc 432, Santa Cruz Biotechnology; PDGFRA 07–276, Upstate, Charlottesville, VA, USA and KIT Ab-3, clone K45, Neomarkers, Fremont, CA, USA) subsequently blotted to a membrane and incubated first with anti p-Tyr antibody (clone 4G, Upstate) and then with antibodies for the specified receptors. As positive controls, for PDGFRA and PDGFRB, an NIH3T3 cell line was employed, whereas for KIT a Δ559 cell line expressing a constitutively activated KIT receptor was used.
